# COVID-19 in Pregnant Women, Maternal—Fetal Involvement, and Vertical Mother-to-Child Transmission: A Systematic Review

**DOI:** 10.3390/biomedicines10102554

**Published:** 2022-10-13

**Authors:** Juan Carlos Sánchez-García, Nuria Pilar Carrascosa Moreno, María Isabel Tovar-Gálvez, Jonathan Cortés-Martín, Antonio Liñán-González, Leticia Alvarado Olmedo, Raquel Rodríguez-Blanque

**Affiliations:** 1 Research Group CTS1068, Andalusia Research Plan, Junta de Andalucía, 41006 Sevilla, Spain; 2 Nursing Department, Faculty of Health Sciences, University of Granada, 18071 Granada, Spain; 3 Canarias University Hospital, Carretera Ofra S/N, 38320 La Laguna, Spain; 4 Nursing Department, Faculty of Health Sciences in Ceuta, University of Granada, 51001 Ceuta, Spain; 5 Nursing Department, Faculty of Health Sciences in Melilla, University of Granada, Melilla Campus, 52005 Melilla, Spain

**Keywords:** COVID-19, SARS-CoV-2, pregnancy, vertical mother-to-child transmission, breast milk, vaccination

## Abstract

Pregnant women are included in the COVID-19 risk groups even if they do not have any pathology. This requires an analysis of research focused on pregnant women to understand the impact of SARS-CoV-2 on their condition. There is also a need to know whether there is vertical mother-to-child transmission, as well as other consequences in case the pregnant woman is infected and COVID-19 positive. A systematic review was carried out to analyze the existing information on the complications of a pregnant woman infected with the SARS-CoV-2 coronavirus and the possibility of vertical transmission from mother to child, registered in the PROSPERO website and searched in the PubMed, Scopus, CINAHL, and Cochrane Library databases. Finally, 22 articles were included in the review. The review suggests that vertical transmission from mother to child could be exceptionally possible at the time of delivery or breastfeeding, but not through the placenta. It is interesting to point out the good acceptance of vaccination by pregnant women, which may be the reason for the low infectivity. Further research on pregnant women should be carried out to provide evidence on vertical mother-to-child transmission and the role of breast milk in relation to SARS-CoV-2.

## 1. Introduction

COVID-19 is a disease caused by a new, already-known virus, called SARS-CoV-2, which caused unexplained pneumonia in late December 2019 and resulted in a global pandemic, which is still present today [1]. Symptoms of the disease can result in a range of symptoms from the need for admission to intensive care units to no symptoms at all. It has been evidenced that it does not act with the same effect on all people and that the most adverse effects appear in those who have some pathology such as respiratory or cardiac diseases [2].

On 11 March, a global pandemic was declared, which meant a great change in the economic, social, and health spheres, negatively affecting vulnerable groups in particular. The people most at risk of developing a serious COVID-19 infection are those over 60 years of age or those with a pathology such as hypertension [3]. Among the groups at risk is the population of pregnant women, whose changing anatomical and physiological status causes alterations that affect their respiratory system, as well as their immune system, cardiovascular function, and even coagulation. Although the risk of contracting the infection is the same as in the general population, their status means that the evolution of the disease may be more severe at the respiratory level [4,5] and may even increase the risk of presenting with pre-eclampsia and alterations at the placental level [6,7,8]. We refer to the risks that the mother may suffer but also the fetus, as vertical mother-to-child transmission may occur [9]. Viral infection during pregnancy can seriously harm the fetus, leading to miscarriage, fetal death, intrauterine growth retardation, or the newborn, such as various types of sequelae [10].

Therefore, pregnant women need their own analysis of the mechanism of transmission from mother to fetus, to explore whether or not it really occurs by vertical mother-to-child transmission and the possible complications that may appear at the time of delivery. It is also important to analyze some aspects of the pathology produced by the SARS-CoV-2 virus and whether immunization by vaccination may have had any influence on this possible vertical mother-to-child transmission, thus producing a document that can help in future research [11,12,13,14]. 

The aim of this systematic review was to analyze whether vertical mother-to-child transmission of the SARS-CoV-2 virus occurs in pregnant women in COVID-19 and what the existing maternal–fetal involvement is.

## 2. Materials and Methods

### 2.1. Review Protocol

The methodology used for the elaboration of this report followed the Preferred Reporting Items for Systematic reviews and MetaAnalyses (PRISMA) review protocol [15], which consists of a 27-point checklist of the most representative sections of an original article, as well as the process of drawing up these guidelines. This systematic review was carried out following a protocol, available on the web: http://www.crd.york.ac.uk/PROSPERO/ (accessed on 5 August 2022), the registration number of which is ID331580.

### 2.2. Eligibility Criteria

We considered articles from 1 December 2019 to 1 March 2022 that provided information on COVID-19 in pregnant women and the possibility of fetal transmission, with no restrictions on the language of publication. We accepted any type of article in line with the topic to be addressed. Two researchers independently assessed all the references identified in the search. First, we screened the references according to the title and abstract. Subsequently, articles that met the inclusion criteria in the first phase were read in full text to determine their final inclusion. In cases where there was disagreement between the two researchers on the inclusion of a manuscript, a third researcher was consulted.

Data on quality, patient characteristics, interventions, and relevant outcomes were obtained independently by the authors.

### 2.3. Sources of Information

The literature search was carried out in the databases Scopus, PubMed, CINAHL, CINAHL, and Cochrane library. A hand search was conducted using reference lists of studies to find other relevant studies. The structured language used was obtained using MeSH terms and Health Sciences (DeCS) descriptors. The descriptors used were “COVID-19”, “SARS-CoV-2”, “pregnancy”, “fetal transmission”, “complications”, and “outcomes”, and the Boolean operators used were AND and OR.

### 2.4. Search Strategy

Table 1 shows the search strategy that was used to carry out this work, together with the date on which the search was carried out.

### 2.5. Risk of Bias in Individual Studies

In order to carry out the methodological evaluation of the articles selected for this study, the design, methodology, and type of study of each paper were analyzed, with the aim of selecting the most specific methodological evaluation scale for each case.

Of the 22 articles, 8 were literature reviews, 8 systematic reviews, 2 cohort studies, 3 case studies, and 1 randomized clinical trial.

Articles with a case study design were assessed using the Rating Scale for Single Participants Designs (SCED). The SCED was constructed including 11 items, of which 10 were used to assess methodological quality and one for the use of statistical analysis. One point was added for each item present, and a maximum score of 11 points could be obtained. Between 9 and 10 indicates very good quality; between 6 and 8 indicates good quality; between 4 and 5 indicates poor quality; and below 4 indicates poor quality. The cut-off point chosen to select the studies was those that obtained a score of 6 points or more.

Table 2 shows the result obtained after applying the methodological evaluation using the SCED scale.

The AMSTAR-2 (A Measurement Tool to Assess Systematic Reviews) methodological assessment scale was used for the reviews [17]. AMSTAR-2 provides a broad assessment of quality, incorporating imperfections that may have arisen due to improper conduct of the review. AMSTAR-2 was constructed to include 16 domains, which present simple response options: “yes” when the product is positive; “no”, if the standard was not met or the existing information was too limited to answer; and “partial yes”, in situations where partial adherence to the standard was given. While not providing an overall rating, four levels of confidence emerge: high, moderate, low, and critically low.

Table 3 below shows the results obtained after applying the methodological evaluation using the AMSTAR-2 scale.

### 2.6. Selection of Studies

**Figure 1:**
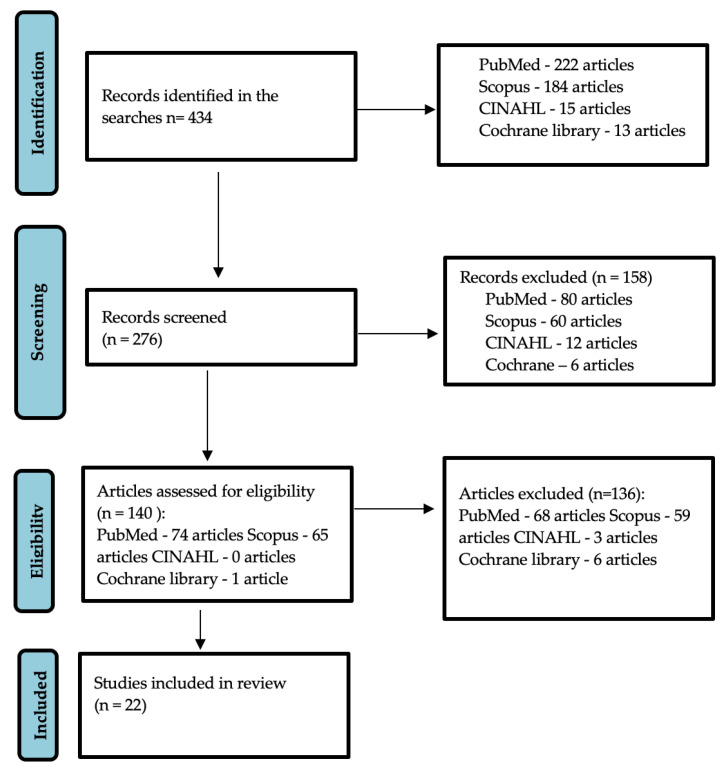
Flow chart (PRISMA guidelines).

A search was carried out in the different databases using a combination of keywords, obtaining a total of 434 documents. The selected studies were published between 2020 and 2022. Duplicates were eliminated, leaving a total of 276 items. In the selection of articles that could be related to the topic to be addressed, the reviewers carried out a selective reading of the title and abstract of 140 papers. Finally, 22 articles were included in the present review, resulting in the definitive study list shown in Figure 1.

## 3. Results

The main characteristics of all the studies included are shown in Table 4.

### 3.1. Complications in Pregnant Women

Several studies have been carried out involving pregnant women who tested positive for COVID-19 after a DTAI (Diagnostic Test for Active Infection), in order to study and learn about the most common complications that occurred in them.

Following data collection, the most common symptoms in pregnant women with COVID-19 were found to be fever, cough, fatigue, dyspnoea, diarrhea, and malaise [10]. As the most common complications in these women, we found viral pneumonia [4] and hypertensive disorders such as pre-eclampsia [10].

Yang et al. [9] in their study found a high rate of pregnant women testing positive for COVID-19 who had to undergo a cesarean section due to respiratory distress and fetal intrauterine distress.

In the study by Saroyo et al. [20], they identified that the virus can cause pre-eclampsia, preterm prelabour rupture of membranes, and fetal growth restriction during pregnancy, resulting in circulatory failure for the mother and sometimes for the fetus.

It has also been observed that the infection of pregnant patients with SARS-CoV-2 may increase the risk of maternal mortality since a number of cases were found in Iran, in which they found severe complications in pregnant women, where 1/9 women became ventilator dependent, 1/9 recovered after a long period of hospitalization, and 7/9 died [26].

In the study by Saroyo et al. [20], 4/7 women were observed, who during the first trimester of pregnancy, suffered a miscarriage, and in the second trimester 6/10 pregnant women with COVID-19 had to be intubated and admitted to the ICU (intensive care unit).

There are several ways to diagnose COVID-19 in pregnant women. Generally, it is detected in test samples collected from saliva, upper and lower respiratory tract secretion, urine, and feces, but blood samples can be considered the most important tool for such diagnosis [12].

It is in blood samples where we can find more exhaustive information about the damage caused by this virus in pregnant women, such as those described by Ferrer–Oliveras et al. [28] in their study, in which they refer to the virus-causing deregulation of the proportion of Th17 cells (T-helper lymphocytes) leading to an increase in T-helper cells.

The literature reviewed indicates that most of the cases in which women suffer spontaneous abortions as a result of COVID-19 are caused by placental insufficiency related to the virus [21]. Ferrer–Oliveras et al. [28] shows that this virus induces states of hypercoagulability, leading to thrombotic–hemorrhagic events in pregnant women.

### 3.2. Fetal/Newborn Complications

To understand the possible complications caused by SARS-CoV-2 at the fetal and neonatal levels, the placenta must first be studied. In their study of the placenta, Resta et al. [19] found the SARS-CoV-2 protein in the placental cells of COVID-19-positive pregnant women. They also found fibrin deposits and inflammatory infiltrates, which produce poor vascular perfusion at the maternal level and growth restriction at the fetal level.

Aghaamoo, Ghods, and Rahmanian [26] report that these fibrin deposits and multiviral infarction in the placenta of pregnant women infected with SARS-CoV-2 may disturb nutrient transport to the fetus. These factors could cause premature contraction and premature rupture of the membrane, causing premature delivery [20].

A study by Ghema et al. [18] showed interesting results in 30 newborns born to COVID-19-positive mothers, with 98% of them being PCR-negative. One of the positives had pneumonia and one died, because of severe sepsis. Although the two subjects had different characteristics and outcomes, a study of their respective placentas showed a similar phenomenon of premature rupture of the membranes. This premature rupture of the membranes can have major neurological repercussions in the life of these subjects such as vasculitis or fetal brain injury [30].

Ghema et al. [18] and Crovetto et al. [35] found through their fetal studies other less frequent complications such as perinatal asphyxia, respiratory failure, multi-organ dysfunction, brain damage, malformation, intrapartum fetal distress, and even death. Another complication in children of COVID-19-infected pregnant women, though rare, is a neonatal inflammatory syndrome whose clinical manifestations are elevated inflammatory biomarkers, organ dysfunction, and in some cases myocardial dysfunction [33].

Based on the studies reviewed, the clinical presentation of the virus in neonates differs from that in adulthood, suggesting that the impact of COVID-19 in neonates may be limited [34]. Short-term outcomes of neonatal infection are positive, but the long-term impact on neurodevelopment is unknown, and the continuous study of these subjects is necessary [30].

### 3.3. Vertical Mother-to-Child Transmission

There is scientific evidence of transplacental transmission of emerging diseases such as HIV and Ebola [29]. Researchers such as Resta et al. [15] have suggested that this may also be the situation with SARS-CoV-2. The placenta is a protective barrier against disease and infection for the fetus and in the case of SARS-CoV-2, it is considered to prevent transmission of maternal viral infection [34], but it has been observed in the review by Barcelós et al. [9] that it is a potential locus of infection for SARS-CoV-2.

Despite the millions of confirmed cases of COVID-19 worldwide, only one case has been found that met the criteria for vertical mother-to-child transmission. A 23-year-old pregnant woman, 35 weeks gestational age, was admitted with fever and severe cough with PCR diagnosis of the virus. She underwent a cesarean section for acute fetal distress and tested positive for amniotic fluid. Nasopharyngeal and rectal swabs were collected and found to be positive. In the first days of life, the newborn presented neurological symptoms and a central nervous system disorder diagnosed by magnetic resonance imaging. The placental examination was positive for SARS-CoV-2, suggesting vertical mother-to-child transmission [35].

It has been observed in placental studies that SARS-CoV-2 protein is present, which could produce serious complications in subjects although when tested for COVID-19 have been negative, indicating that no intrauterine transmission has occurred [19]. Jamieson and Rasmussen [31] in their review show that intrauterine transmission is a rare event and is very unusual. However, some newborns after taking the DTAI (Diagnostic Test for Active Infection) for COVID-19 were positive, but it was unknown whether the infection was before, during, or after birth through close contact with an infected person [24].

Khedmat et al. [22] report that in some newborns of positive mothers, high levels of Immunoglobulin M have been found within 2 h of birth, suggesting that in some cases an in utero infection with this virus is possible. There are reassuring results which indicate that vertical mother-to-child transmission rarely affected mortality and had a favorable evolution [32].

Studies were also conducted on the breast milk of those mothers who were infected, as breast milk could be a mechanism of transmission to the neonate; however, these indicated that it was the close contact of the neonate with its mother that caused the infection, as no evidence of SARS-CoV-2 RNA or protein was found in breast milk [27]. However, studies suggest that the mechanisms involved in maternal–fetal transmission are unclear and that transmission is probable, but the incidence is extremely low [33] and most are acquired at delivery or postpartum [25].

**Table 4:** Table of results.

Author	Objectives	Conclusions
Wang et al. (2021) [21]	To review the clinical manifestations, and maternal and perinatal outcomes of COVID-19 during pregnancy.	There is no evidence of fetal transmission and infants and children experience only mild forms of COVID-19.
Diriba, Awulachew, and Getu (2020) [4]	Assess the effect of coronavirus infection during pregnancy and the possibility of vertical mother-to-child transmission.	None of the studies reported transmission of CoV from mother to fetus in utero, which may be due to a very low expression of angiotensin-2 converting enzyme in early cells at the maternal-fetal interface.
Khedmat et al. (2021) [22]	To study the potential for vertical mother-to-child transmission of COVID-19 in pregnant women. Summary of symptoms and clinical outcomes in mothers and babies, as well as proposed therapies and preventive health solutions.	Babies with respiratory problems may be born to some COVID-19 positive mothers.
Robaina–Castellanos and Riesgo–Rodríguez (2021) [23]	To summarize and analyze the published evidence on the modes of vertical mother-to-child transmission of SARS-CoV-2 (intrauterine or intrapartum).	Congenital and intrapartum SARS-CoV-2 infection in the fetus or newborn is possible, but rare.
Fernandez–Perez et al. (2021) [16]	To inform about how SARS-CoV-2 acts in the body, as well as the imaging studies that help to diagnose it.	It is a virus belonging to the coronavirus family. It affects the respiratory tract and has other adverse side effects that are still under study.
Yang et al. (2020) [12]	Retrospective cohort study conducted in Wuhan with the aim of finding out the most adverse effects that COVID-19 can produce.	This study shows that the two most common adverse effects experienced by pregnant women are premature delivery and cesarean delivery.
Kazemi et al. (2021) [24]	Systematic review aimed at understanding the factors that occur in women who had an abortion after infection.	One factor causing miscarriage may be inflammation of the placenta.
Ribeiro et al. (2021) [25]	To inform about some of the aspects of the pathology.	No specific changes were found in the placentas of pregnant women.
	Placenta in SARS-CoV-2 infection.	The findings show poorer maternal and fetal perfusion in them than in non-pregnant women.
Aghaamoo, Ghods, and Rahmanian (2021) [26]	Investigate possible undesirable maternal and fetal-neonatal consequences of COVID-19.	Detection and follow-up of infected pregnant women reduces the risk of maternal and neonatal death and provides control over complications.
Gratacós et al. (2021) [35]	A population-based study to describe the impact of SARS-CoV-2 infection on pregnancy outcomes.	The rate of pregnancy complications in infected women was similar to that of non-pregnant women.
Ghema et al. (2021) [18]	Study examining 30 newborns of COVID-19 positive women with the aim of providing documented information on mother-to-child transmission.	Mother-to-fetal transmission of the virus was not detected in most of the reported cases, although they were detected positive by PCR.
Kant et al. (2021) [27]	Systematically synthesize the available literature on various modes of transmission (congenital, intrapartum and postpartum), clinical features and outcomes of infection.	Limited evidence suggests that the risk of SARS-CoV-2 infections in newborns is extremely low and postpartum acquisition was the most common mode of infection in newborns.
Ferrer–Oliveras et al. (2021) [28]	This document provides information on research to elucidate potential harmful responses to SARS-CoV-2 and other coronavirus infections.	A severe form of COVID-19 is an immune-mediated hyperinflammatory disorder triggered by a viral infection.
Barcelos et al. (2021) [9]	An evaluation of the available evidence on vertical mother-to-child transmission of severe acute respiratory syndrome coronavirus 2 (SARS-CoV-2).	The risk of vertical mother-to-child transmission of SARS-CoV-2 is very low. Despite the thousands of pregnant women who have been affected, the sample is not sufficient to create the evidence, so further research is needed.
Auriti et al. (2021) [29]	Collects information on possible harm to the fetus in the event of transmission of infection, as well as diagnostic testing.	When transmission of a viral infection occurs, the fetus or newborn may not have any adverse effects at the time, but in the long term it may.
Cavalcante et al. (2021) [30]	Obstetric outcomes of COVID-19 positive women and possible risks.	Possible risk of neurological damage in children of infected women.
Jamieson and Rasmussen (2022) [31]	Update on COVID-19 in pregnancy.	With the development of the vaccine, it has been studied that it is favourable for both mother and fetus and helps to protect both.
Resta et al. (2021) [19]	A case–control study performed in order to highlight any histopathological alterations.	The research demonstrated fetal endothelial distress, as well as the presence of particles attributable to SARS-CoV-2.
Yoon, Hang, and Ahn (2020) [32]	To assess the clinical manifestations and outcomes of newborns of women who had coronavirus 2019 disease during pregnancy.	Evidence suggests that the virus rarely causes fetal and neonatal mortality.
Morrison et al. (2022) [33]	Gathering information on the care that can be provided to the pregnant woman, as well as treatments to treat COVID-19 without harming the fetus.	Pregnant women are very vulnerable to drugs and therefore further research is needed to find out which drugs will not cause any harm to the mother or fetus.
Ryan et al. (2022) [34]	Review of the current available evidence related to the impact of the COVID-19 pandemic on newborns, the effects on their health, the impact on quality of care and indirect influences on their clinical course.	The evidence should be used to continue to promote best practice in neonatal care.
Saroyo et al. (2021) [20]	To know the effect of Remdesivir in pregnant women with COVID-19 and how it influences their recovery.	The Remdesivir protocol for pregnant women with moderate to severe COVID-19 symptoms has resulted in favourable clinical improvement with a shorter recovery period and no adverse effects during the hospitalization period.

## 4. Discussion

Firstly, it is interesting to highlight that the data on the incidence of cesarean surgeries in pregnant that were positive in the test increased considerably, because they presented respiratory difficulty and developed pre-eclampsia. Thus, these symptoms produced a risk for both the woman and the fetus, confirming that there is a direct relationship between COVID-19 and complications in pregnancy, even if the symptoms presented are not severe [12,17,20,26].

After delivery, the placenta was studied independently and interesting results were obtained, as there were findings of altered coagulation factors in the premature rupture of the placenta, a question related to the alteration in coagulation produced by COVID-19 [12,34,36]. 

As for vertical mother-to-child transmission, it appears that the placenta continues to act as a barrier as with other viruses, as there is little likelihood of it occurring. In the few studies that provide data from fetuses testing positive, it is not known exactly whether it occurs before, during, or after birth. Breast milk showed no trace of SARS-CoV-2 proteins, so this does not appear to be a mechanism of transmission [22,24,27,31,32,33]. There are few studies that provide data on the impact of COVID-19 on the fetus, but it appears to be limited as non-specific infectivity test results have been reported. It would be interesting to focus on this topic by relating both maternal vaccination and subsequent breastfeeding as possible protective factors [14,30,34,35,37]. One of the reasons for mother-to-child transmission is the close contact between positive mothers and newborns during breastfeeding, so it can be transmitted through breathing. Therefore, they recommend that all COVID-19-positive mothers practice adequate hand hygiene and pump milk so that the baby can be fed by a healthy caregiver to prevent transmission of the virus. Mothers who prefer skin-to-skin contact should consider the consequences of this, and adopt excellent hand washing and the use of surgical masks to prevent the transfer of Flügge droplets [9,10,34].

Authors such as Wang et al. [21], Robaina–Castellanos et al. [23], and Crovetto et al. [35] focused on finding information about the complications that COVID-19 could cause in pregnant women and at the fetal level, and whether or not there was a possibility of vertical mother-to-child transmission. It has been observed that pregnant women are more likely to be susceptible to SARS-CoV-2 infection than women who are not pregnant, because their immune systems change as a result of pregnancy and respond differently to SARS-CoV-2 [35,38].

The most common COVID-19-related problems in pregnant women are those affecting the respiratory system and blood pressure, leading to hypertensive disorders such as pre-eclampsia [39,40]. Those problems that affect the respiratory system are viral pneumonia in the first place, requiring urgent admission to the intensive care unit, in addition to the fact that in pregnancy there is a 20% increase in the demand for oxygen and at the same time the residual capacity decreases [24]. This respiratory insufficiency can lead to an interruption of the placental flow and cause a miscarriage [34,36]. This problem not only affects the mother, but can also have an impact on the fetus, causing serious complications in its development and nervous system, as seems to be the pattern in adults [29,39,41]. 

With respect to treatment for infected pregnant women being a challenge compared to the general population, a study was conducted in which five cases of pregnant women treated with Remdesivir (RNA polymerase inhibitor) during hospitalization were studied. All cases showed a shorter duration in hospital with rapid improvement of clinical symptoms and no adverse effects. Although the study showed good results, it has not yet been established as a standardized therapy for treating pregnant women with COVID-19, as its effectiveness needs to be further studied [12,17,42]. Three COVID-19 vaccines are currently available, two mRNA vaccines (Pfizer and Moderna) and one adenoviral vector vaccine (Johnson & Johnson). Although any of the currently licensed vaccines can be administered to pregnant women, the SEGO (Spanish Society of Gynaecology and Obstetrics) states its preference for the mRNA vaccine because there are more safety data than for the adenoviral vaccine [5,12,43]. Furthermore, the administration of vaccines to a breastfeeding woman poses no risk to either her or her child as they do not contain live microorganisms and therefore have no infective capacity [14,30,31].

During the peak of the pandemic, there was an increase in hospitals performing cesarean sections. This was because most pregnant women had respiratory compromise caused by the infection which would complicate delivery while causing fetal distress, intrauterine growth restriction, or even death [24,31]. The causative factors of fetal loss in the first weeks of gestation are mostly due to inflammatory events affecting the placenta [19,26]. This may cause premature contraction and rupture of the membrane leading to premature delivery and stillbirth [18,23].

The fact that there are neonates showing damage caused by the virus raises suspicions of possible vertical transmission from mother to child. Since the beginning of the pandemic, the vertical mother-to-child transmission of this virus has been debated, with some studies denying this fact and others not ruling out the idea due to the detection of antibodies in the umbilical cord blood of newborns [24,27,29,30]. It would be interesting to be able to perform studies focusing on the analysis of antibodies in cord blood of placental tissue, amniotic fluid, umbilical cord blood, and neonatal blood in the first 12 h of life, in addition to a PCR test using a nasopharyngeal swab [34].

There is no antiviral treatment for COVID-19 in newborns and therefore guidelines for the management of this type of patient should be established, as well as for pregnant women [44]. Although pregnant women are included in the general adult population by age, as was the case in previous pandemics, they used to be excluded from these vaccination programs. However, they are a population group that tends to accept vaccination well, for themselves and their children, so future studies focusing on this population group would promote better adherence to vaccination because of possible reluctance to immunize them against SARS-CoV-2 virus [13,14,45,46,47], since recent studies show the safety of the administration of the vaccine against COVID-19, with no adverse effects appearing in pregnancy after administration, showing the clear risk of non-vaccination during this period [48,49,50,51,52,53].

## 5. Conclusions

Pregnancy increases the risk of severe COVID-19 disease, but the patterns of COVID-19 that affect some people more and others less are not yet known, although adverse fetal outcomes are more associated with women with severe complications.

The analyzed research concludes that the risk of vertical mother-to-child transmission of SARS-CoV-2 from mother to fetus is very low, and is considered a rare but possible event, although more studies focused on this population will be needed to consider it as evidence.

## Figures and Tables

**Table 1:** Search strategies.

Sources of Information	Search String
PUBMED	Search: (COVID-19) OR (SARS- CoV-2) AND (pregnancy) AND (fetal transmission) AND (complications) AND (outcomes). Filters: Full text, publication date 5 years.
SCOPUS	Search within (article title, abstract, keywords): (COVID-19) OR (SARS-CoV-2) AND (pregnancy) AND (fetal transmission) AND (complications) AND (outcomes) AND (Limit-to (DOCTYPE “ar”)).
COCHRANE LIBRARY	Search: (COVID-19) OR (SARS- CoV-2) AND (pregnant) AND (outcomes) in title, abstract, keyword.
CINAHL	Search: (COVID-19) OR (SARS- CoV-2) AND (pregnant) AND (rct) AND (outcomes) AND (vertical mother-to-child transmission).

**Table 2:** Methodological assessment according to the SCED scale.

Author	Article	Numerical Score
Fernandez–Perez et al. [16]	SARS-CoV-2: What it is, how it acts, and how it manifests in imaging studies.	8
Yang et al. [17]	Pregnant women with COVID-19 and risk of adverse birth outcomes and maternal-fetal vertical mother-to-child transmission: A population-based cohort study in Wuhan, China.	6
Ghema et al. [18]	Outcomes of newborns to mother with COVID-19.	6
Resta et al. [19]	SARS-CoV-2 and placenta: New insights and perspectives.	8
Saroyo et al. [20]	Remdesivir treatment for COVID-19 in pregnant patients with moderate to severe symptoms: Serial case report.	7

**Table 3:** Methodological assessment according to the AMSTAR-2 scale.

Author	Article	Assessment of Overall Confidence
Wang et al. [21]	Impact of COVID-19 on pregnancy.	High
Diriba et al. [4]	The effect of Coronavirus infection (during pregnancy and the possibility of vertical maternal–fetal transmission: A systematic review.	High
Khedmat et al. [22]	Pregnant women and infants against the infection risk of COVID-19. A review of prenatal and postnatal symptoms.	Moderate
Robaina–Castellanos et al. [23]	Congenital and intrapartum SARS-CoV-2 infection in neonates, hypotheses, evidence, and perspective.	High
Kazemi et al. [24]	COVID-19 and cause of pregnancy loss during the pandemic: A systematic review.	Moderate
Ribeiro et al. [25]	SARS-CoV-2 infection and placental pathology infection.	Moderate
Aghaamoo, Ghods, and Rahmanian [26]	Pregnant women with COVID-19 the placental involvement and consequences.	Moderate
Kant et al. [27]	Clinical features and outcome of SARS-CoV-2 infection in neonates: A systematic review.	Moderate
Ferrer––Oliveras et al. [28]	Immunological and physiopathological approach of COVID-19 in pregnancy.	Moderate
Barcelos et al. [9]	Vertical mother-to-child transmission of SARS-CoV-2: A systematic review.	Moderate
Auriti et al. [29]	Pregnancy and viral infections: Mechanisms of fetal damage diagnosis and prevention oof neonatal adverse outcomes from cytomegalovirus to SARS-CoV-2.	High
Cavalcante et al. [30]	Maternal immune responses and obstetrical outcomes of pregnant women with COVID-19 and possible health risks of offspring.	Moderate
Jamieson and Rasmussen [31]	An update on COVID-19 and pregnancy.	High
Yoon, Hang, and Ahn [32]	Clinical outcomes of 201 neonates born to mothers with COVID-19: A systematic review and meta-analysis.	High
Morrison et al. [33]	COVID-19: Can we treat the mother without harming her baby?	Moderate
Ryan et al. [34]	Neonates and COVID-19: State of the art neonatal sepsis series.	Moderate

## Data Availability

Not applicable.
